# Oral Valganciclovir as a Preemptive Treatment for Cytomegalovirus (CMV) Infection in CMV-Seropositive Liver Transplant Recipients

**DOI:** 10.1371/journal.pone.0123554

**Published:** 2015-05-05

**Authors:** Jong Man Kim, Choon Hyuck David Kwon, Jae-Won Joh, Young Eun Ha, Dong Hyun Sinn, Gyu-Seong Choi, Kyong Ran Peck, Suk-Koo Lee

**Affiliations:** 1 Department of Surgery, Samsung Medical Center, Sungkyunkwan University School of Medicine, Seoul, Republic of Korea; 2 Division of Infection, Department of Medicine, Samsung Medical Center, Sungkyunkwan University School of Medicine, Seoul, Republic of Korea; 3 Division of Gastroenterology, Department of Medicine, Samsung Medical Center, Sungkyunkwan University School of Medicine, Seoul, Republic of Korea; The University of Hong Kong, HONG KONG

## Abstract

**Objectives:**

Cytomegalovirus (CMV) infections in liver transplant recipients are common and result in significant morbidity and mortality. Intravenous ganciclovir or oral valganciclovir are the standard treatment for CMV infection. The present study investigates the efficacy of oral valganciclovir in CMV infection as a preemptive treatment after liver transplantation.

**Methods:**

Between 2012 and 2013, 161 patients underwent liver transplantation at Samsung Medical Center. All patients received tacrolimus, steroids, and mycophenolate mofetil. Patients with CMV infection were administered oral valganciclovir (VGCV) 900mg/day daily or intravenous ganciclovir (GCV) 5mg/kg twice daily as preemptive treatment. Stable liver transplant recipients received VGCV.

**Results:**

Eighty-three patients (51.6%) received antiviral therapy as a preemptive treatment because of CMV infection. The model for end-stage liver disease (MELD) score and the proportions of Child-Pugh class C, hepatorenal syndrome, and deceased donor liver transplantation in the CMV infection group were higher than in the no CMV infection group. Sixty-one patients received GCV and 22 patients received VGCV. The MELD scores in the GCV group were higher than in the VGCV group, but there were no statistical differences in the pretransplant variables between the two groups. AST, ALT, and total bilirubin levels in the GCV group were higher than in the VGCV group when CMV infection occurred. The incidences of recurrent CMV infection in the GCV and VGCV groups were 14.8% and 4.5%, respectively (P=0.277).

**Conclusion:**

Oral valganciclovir is feasible as a preemptive treatment for CMV infection in liver transplant recipients with stable graft function.

## Introduction

Cytomegalovirus (CMV) infections in liver transplant recipients are one of the most common infectious complications and result in significant morbidity and mortality. The incidence of CMV infection in CMV seropositive donors and seropositive recipients (CMV D+/R+) is approximately 23% to 85% after LT, and 15% to 20% of these recipients develop CMV disease [1–3].

CMV infection has direct and indirect effects in liver transplant recipients [4]. CMV disease as a direct effect of CMV infection is a risk factor for graft and patient survival in liver transplant recipients with CMV D+/R+ [3]. The indirect effects of CMV infection in liver transplant recipients are chronic rejection, interaction with hepatitis C virus (HCV), HCV recurrence, posttransplant lymphoproliferative disorder, and diabetes [5]. Intravenous ganciclovir or oral valganciclovir are the standard treatment for CMV infection.

Universal prophylaxis and preemptive treatment are acceptable for the prevention of CMV disease [6]. A previous pharmacokinetic study showed similar drug exposure to ganciclovir after a single oral dose of 900 mg valganciclovir (VGCV) as compared to an intravenous dose of 5 mg/kg ganciclovir (GCV) [7]. Recently, oral VGCV and intravenous GCV were shown to have similar efficacy in pre-emptive CMV treatment in solid organ transplant recipients [8, 9]. So far, no data are available on the efficacy of 900 mg VGCV once daily as compared to intravenous 5 mg/kg ganciclovir twice daily in the preemptive therapy of CMV infection in liver transplant recipients, despite the fact that oral VGCV is commonly used as antiviral prophylaxis or as a preemptive treatment in liver transplant recipients [10].

We have compared the clinical characteristics between patients with and without CMV infection after transplantation and investigated the efficacy of oral VGCV in CMV infection as a preemptive treatment after CMV D+/R+ adult liver transplantation.

## Materials and Methods

### Patients

We reviewed the medical records of 161 consecutive adult liver transplant cases between May 2012 and September 2013 at Samsung Medical Center, Seoul, Korea. All patients were CMV donor positive/recipient positive. Patients younger than 18 years of age, patients who died within one month of liver transplantation, the presence of CMV disease, cyclosporine use as maintenance therapy after liver transplantation, and CMV seropositive donor/recipient negative (CMV D+/R-) status were excluded. All medical records of all patients were reviewed for epidemiologic and clinical characteristics. All patients were followed until death or the end of the study in October 2014. The following data were collected before CMV infection detection: patient demographics, preoperative diagnosis, Child-Turcotte-Pugh (CTP) scores, Model for End-stage Liver Disease (MELD) scores, type of liver transplantation, ABO-incompatibility, and retransplantation. Serum creatinine was measured by Rate-Blanked kinetic alkaline picrate using Roche Modular system. When CMV infection was diagnosed, laboratory findings, the highest number of pp65-staining cells, the time from liver transplantation to CMV infection, and the duration of antiviral agents use were noted. Our study was approved by the Institutional Review Board of Samsung Medical Center in Seoul (SMC 2014-07-088). Consents from patients were not obtained because our study was retrospective design. Thus, our study approved in IRB. All patients' information and records were de-identified prior to analysis (S1 File).

### Virologic Follow-up and CMV Infection Treatment

After liver transplantation, all patients were routinely tested for CMV using a CMV pp65 antigenemia assay. We did not routinely use antiviral prophylactic therapy in the liver transplant recipients. We selected a preemptive treatment strategy using intravenous GCV to prevent CMV-related complications. For the first month after transplantation, EDTA-treated blood samples were examined weekly for the presence of CMV. Testing was performed three times a week if the patient had a known CMV infection. In the absence of symptoms, patients were routinely monitored for CMV once a month. If a patient had an unexplained fever or if a CMV infection was clinically suspected, a CMV antigenemia assay was conducted [3].

CMV infection was defined as a CMV pp65 antigen-positive cell number greater than ten positive cells per 400,000 white blood cells. When CMV infection was diagnosed, the patient was admitted to the hospital and preemptive treatment was initiated regardless of clinical manifestations. CMV assays were conducted three times a week until a negative result was obtained. Preemptive antiviral therapy consisted of intravenous GCV and oral VGCV. Intravenous GCV was administered daily for 10 to 14 days until a negative CMV assay was confirmed. In patients with stable liver graft function, oral VGCV (900mg/day) was administered daily for 2 or 3 weeks until a negative CMV assay was confirmed. The dose of intravenous GCV and oral VGCV were adjusted according to patient creatinine clearance.

### Immunologic Regimens

Tacrolimus, steroids, and mycophenolate mofetil (MMF) were the primary agents used for immunosuppression after liver transplantation. All transplant recipients were given 500 mg of intravenous methylprednisolone during the anhepatic phase until postoperative day two, this was tapered to 60 mg per day for a period of five days, and then administered at 8 mg, twice per day, for one month starting on postoperative day eight. Tacrolimus treatment was started on postoperative day three, and the optimal blood level was adjusted to maintain a trough plasma concentration of 10–15 ng/mL during the first month and was reduced to 5–10 ng/mL thereafter. MMF was used in combination with tacrolimus and steroids. Starting on postoperative day one, 750 mg MMF was administered twice a day [3].

### Statistical Analysis

Categorical data was compared using a chi-square or Fisher’s exact tests and the Mann-Whitney U test was used for continuous variables. Pretransplant factors for CMV infection were identified with logistic regression analysis. Overall graft and patient survival between groups was determined by Kaplan-Meier methods and differences assessed by the log-rank test. We confirmed the assumption that the hazard ratio in graft and patient survival curves between groups was equivalent prior to analysis. The Statistical Package for the Social Sciences (SPSS) version 21 for Windows was used for all tests. P-values less than 0.05 were considered statistically significant.

## Results

### Baseline characteristics between patients with and without CMV infection

CMV infection developed in 83 patients (51.6%). The baseline characteristics of these patients are summarized in Table 1. There were no statistical differences in gender, age, body mass index, and ABO-incompatibility between the two groups. The proportion of Child-Pugh class C in patients with CMV infection was higher than in patients without CMV infection (51.8% vs. 24.7%), and the median MELD score in patients with CMV infection was higher than in patients without CMV infection (13 vs. 18, P<0.001). The incidences of retransplantation and deceased donor liver transplantation in patients with CMV infection were higher than in patients without CMV infection (P = 0.029 and P<0.001, respectively). Additionally, intensive care unit (ICU) and hospitalization stay were longer in patients with CMV infection than in patients without CMV infection (P<0.001 and P = 0.002, respectively). Multivariate analysis showed that the MELD score (odds ratio, 1.016; 95% CI, 1.020–14.104; P = 0.013) as a pretransplant factor was closely associated with CMV infection.

**Table 1:** Baseline characteristics of patients with and without CMV infection.

	No CMV infection (n = 78)	CMV infection (n = 83)	P-value
Gender—male	65 (84.4%)	65 (78.3%)	0.418
Age (years)	53 (25–69)	53 (22–77)	0.846
BMI	24.4 (17.1–39.8)	24.8 (15.5–36.8)	0.706
CTP			<0.001
A	34 (44.2%)	20 (24.1%)	
B	24 (31.2%)	20 (24.1%)	
C	19 (24.7%)	43 (51.8%)	
MELD score	13 (6–42)	19 (6–54)	<0.001
ABO-incompatible	10 (12.8%)	11 (13.3%)	0.935
Retransplantation	0 (0%)	6 (7.2%)	0.029
DDLT	11 (14.1%)	33 (39.8%)	<0.001
ICU stay (days)	6 (3–14)	6 (4–102)	<0.001
Hospitalization (days)	23 (21–84)	36 (18–141)	<0.001
Follow-up duration (months)	16.9 ± 6.2	18.4 ± 7.4	0.002

CMV, cytomegalovirus; BMI, body mass index; MELD, model for end-stage liver disease; DDLT, deceased donor liver transplantation; ICU, intensive care unit

### Clinical characteristics between intravenous GCV and oral VGCV

Among the 83 patients with CMV infection, twenty-two patients (26.5%) were treated with oral VGCV as a preemptive treatment. Sixty-one patients (73.5%) received intravenous GCV. The median age in the oral VGCV group was younger than in the GCV group (47 vs. 55, P = 0.020) and the median MELD score in the oral VGCV group was lower than in the GCV group (13 vs. 22, P = 0.015). Similarly, the proportion of Child-Pugh class C in oral VGCV group was lower than in intravenous GCV group (36.4% vs. 57.4%). However, there were no statistical differences in gender, body mass index, Child-Pugh class, ABO-incompatibility, retransplantation, deceased donor liver transplantation, and ICU stay between the two groups. The median hospitalization time of the oral VGCV group was shorter than that of the intravenous GCV group (27 vs 41, P<0.001) (Table 2).

**Table 2:** Clinical characteristics of patients who received intravenous GCV and oral VGCV.

	Intravenous GCV (n = 61)	Oral VGCV (n = 22)	P-value
Gender—male	49 (80.3%)	16 (72.7%)	0.548
Age (years)	55 (25–77)	47 (22–67)	0.020
BMI	24.9 (15.5–36.2)	24.3 (20.1–36.8)	0.926
CTP			0.072
A	12 (19.7%)	8 (36.4%)	
B	14 (23.0%)	6 (27.3%)	
C	35 (57.4%)	8 (36.4%)	
MELD score	22 (6–50)	13 (6–54)	0.015
ABO-incompatible	6 (9.8%)	5 (22.7%)	0.150
Retransplantation	6 (9.8%)	0 (0%)	0.334
DDLT	24 (39.3%)	9 (40.9%)	0.898
ICU stay (days)	6 (4–102)	6 (4–9)	0.050
Hospitalization (days)	41 (34–141)	27 (18–48)	<0.001
Follow-up duration (months)	20 ± 8	23 ± 5	0.164

GCV, ganciclovir; VGCV, valganciclovir; BMI, body mass index; MELD, model for end-stage liver disease; DDLT, deceased donor liver transplantation; ICU, intensive care unit

### Differences between the intravenous GCV group and the oral VGCV group

The median hemoglobin level in the oral VGCV group was higher than in the intravenous GCV group (10.8 mg/dL vs 9.1 mg/dL, P = 0.003). The serum total bilirubin, AST, and ALT levels in the oral VGCV group were lower than those in the intravenous GCV group because only patients with stable liver graft received oral VGCV as preemptive treatment. There were no statistically significant differences in white blood cells, platelet counts, INR, albumin, ALP, creatinine, and concentration of FK506 between the two groups (Table 3).

**Table 3:** Clinical characteristics between intravenous GCV and oral VGCV.

	Intravenous GCV (n = 61)	Oral VGCV (n = 22)	P-value
White blood cells (/ul)	6,550 (1,280–27,360)	7,430 (2,410–14,750)	0.871
Hemoglobin (g/dL)	9.1 (4.0–15.9)	10.8 (8.2–14.1)	0.003
Platelet (/ul)	108,500 (21,000–465,000)	155,000 (39,000–329,000)	0.321
Lymphocyte (%)	5.9 (1.4–22.4)	5.7 (2.1–48.9)	0.662
INR	1.17 (0.93–5.51)	1.11 (0.98–1.21)	0.065
Albumin (g/dL)	3.1 (2.5–3.9)	3.2 (2.9–3.9)	0.140
Total bilirubin (mg/dL)	2.2 (0.4–14.0)	0.7 (0.3–2.1)	<0.001
AST (IU/L)	34 (11–445)	21 (11–37)	0.008
ALT (IU/L)	87 (15–959)	30 (15–277)	0.016
ALP (IU/L)	92 (39–391)	86 (35–121)	0.072
Creatinine (mg/dL)	0.98 (0.25–4.13)	0.82 (0.35–2.17)	0.137
Concentration of FK506 (ng/mL)	6.6 (0.5–16.7)	6.2 (1.4–10.8)	0.586
Time from LT to CMV infection (days)	21 (1–221)	30 (9–53)	0.001
Highest titer of CMV antigenemia	22 (10–462)	24 (12–754)	0.313
Duration of antiviral agents use (days)	12 (8–27)	16 (14–21)	0.076

GCV, ganciclovir; VGCV, valganciclovir; AST, aspartate transaminase; ALT, alanine transaminase; ALP, alkaline phosphatase; LT, liver transplantation; CMV, cytomegalovirus

The median time from liver transplantation to CMV infection in the intravenous GCV group was shorter than in the oral VGCV group (21 days vs. 30 days, P = 0.001). The median duration of antiviral agent was 12 days (range, 8–27 days) in intravenous GCV group and 16 days (range, 14–21 days) in oral VGCV group. However, highest titer of CMV antigenemia and duration of antiviral agent use between the intravenous GCV and oral VGCV group did not reach significant levels.

### Outcomes

**Fig 1:**
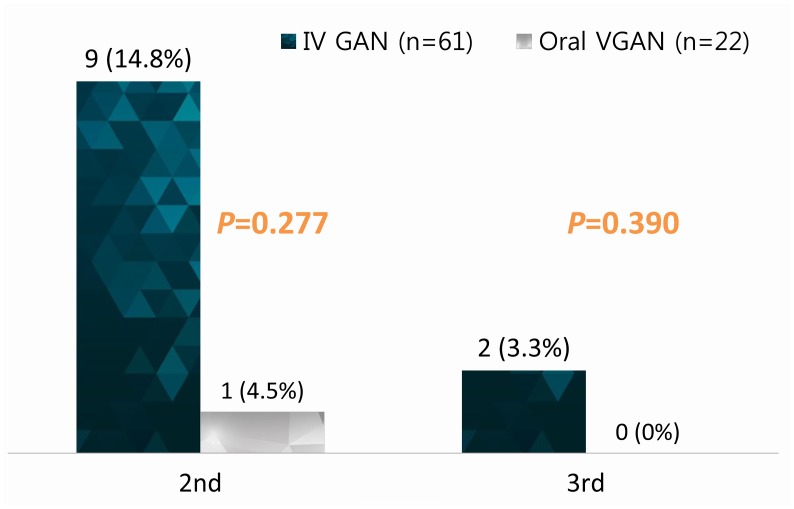
Rates of CMV reinfection after preemptive treatments. Relapsed secondary CMV infection rates in the intravenous GCV and in the oral VGCV groups were 14.8% and 4.5%.

**Fig 2:**
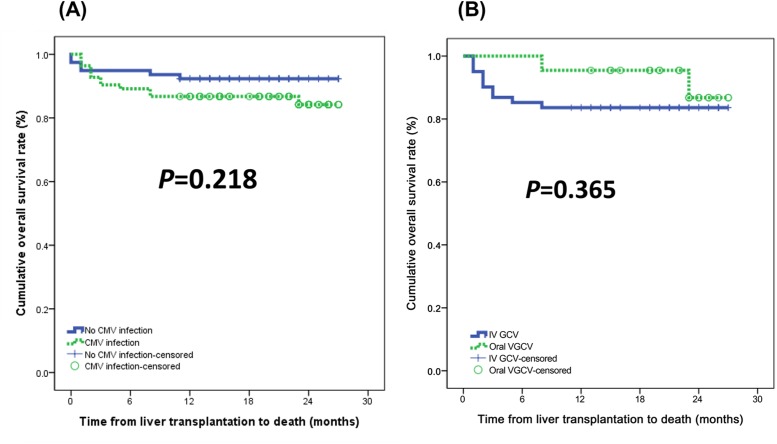
(A) Graft survival and (B) patient survival stratified according to antiviral agent use. The 1-year and 2-year survival rates of patients with CMV infection and patients without CMV infection were 86.7%% vs. 92.3% and 84.2% vs. 92.3%, respectively (*P* = 0.218). With respect to antiviral agents, the 1-year and 2-year survival rates of the intravenous GCV and the oral VGCV groups were 95.5% vs. 83.6% and 86.8% vs. 83.6%, respectively (*P* = 0.365)

None of the patients in either group developed CMV disease. Relapsed secondary CMV infection rates in the intravenous GCV and in the oral VGCV groups were 14.8% (n = 9) and 4.5% (n = 1). There was no statistically significant difference between the two groups (Fig 1). The 1-year and 2-year survival rates of patients with CMV infection and patients without CMV infection were 86.7%% vs. 92.3% and 84.2% vs. 92.3%, respectively (Fig 2(A)). Differences in the overall survival curves between the two groups did not reach a significant level (P = 0.218). With respect to antiviral agents, the 1-year and 2-year survival rates of the intravenous GCV and the oral VGCV groups were 95.5% vs. 83.6% and 86.8% vs. 83.6%, respectively. There was no statistically significant difference in patient survival between the intravenous GCV group and the oral VGCV group (P = 0.365) (Fig 2(B)).

## Discussion

Universal prophylaxis and preemptive treatment are acceptable for the prevention of CMV disease [6]. CMV disease can be serious, rapidly progressive, and fatal. Intravenous GCV has been the standard treatment for patients with suspected CMV disease such as CMV syndrome or tissue-invasive CMV disease. However, intravenous GCV is somewhat inconvenient for patients because it requires frequent hospitalization and long-term intravenous catheter access. Valganciclovir (VGCV) was shown to be effective for the treatment for CMV infection in solid organ transplantation [11]. The advantage of VGCV is that it might benefit patients with regard to convenience and cost.

This study demonstrated that preemptive treatments with oral VGCV and intravenous GCV are equally effective in preventing CMV disease in liver transplant recipients. Preemptive treatment of CMV infection in liver transplant recipients with either oral VGCV or intravenous GCV led to a similar usage of antiviral agents, but the hospitalization stays in the intravenous GCV group were shorter than in the oral VGCV group. In the intravenous GCV group, nine patients developed CMV reinfection. However, only one patient developed CMV reinfection in oral VGCV group. There was, however, no statistically significant difference in CMV reinfection between the two groups. No patients suffered antiviral treatment failure, and no CMV disease developed in present study. The vast majority of recurrent CMV infection is due to ganciclovir-susceptible viruses, and these viruses should remain susceptible and respond well to retreatment with oral valganciclovir or intravenous ganciclovir [12].

A previous study reported that liver transplant recipients showed higher rates of CMV disease and CMV tissue-invasive disease when treated with oral VGCV (19% and 14%, respectively) versus intravenous ganciclovir (GCV; 12% and 3%, respectively) [13]. On the basis of these results, the Food and Drug Administration did not approve VGCV for use in high-risk liver transplant recipients. Re-analysis of the PV16000 trial showed that the risk of CMV tissue-invasive disease in liver transplant recipients was 4.5 times higher with oral VGCV versus intravenous GCV treatment (P = 0.04) [14]. These results suggested that oral VGCV is not a suitable agent for CMV prophylaxis after liver transplantation.

The conversion of VGC to GCV may be decreased in the early posttransplant period due to an esterase deficiency secondary to temporary hepatic dysfunction, bowel dysfunction, or both [13]. In addition, MMF requires the liver esterase for its conversion to mycophenolic acid [13]. These findings suggest the possibility that competition between MMF and oral VGC for the same liver esterase may increase esterase deficiency. Therefore, we have used an oral VGCV only in liver transplant recipients with stable graft function.

In our study, differences in hematological toxicity and gastrointestinal complications between the oral VGCV and the intravenous GCV groups could not be adequately assessed. Further evaluation of side effects in the two treatment groups is warranted prospectively.

Oral VGCV became available in our institution in 2010; however, oral VGCV is not covered by National Insurance Program in Korea. Oral VGCV was used as preferred primary treatment of asymptomatic patients only limited to approval by patients with stable liver graft. In case where approval was not acquired or in cases of co-morbidity requiring hospitalization, intravenous ganciclovir was administered, irrespective of whether these patients had stable liver grafts. However, we do not expect that this possible bias influenced our results because the baseline CMV viral loads and the duration of antiviral agents use in the intravenous GCV and oral VGCV groups were similar, indicating similar CMV activity [3].

In conclusion, our results demonstrate that oral VGCV is equally effective in the preemptive treatment of CMV infection following liver transplantation. Patients in the oral VGCV group had stable liver graft function, and our study does not necessarily suggest the conclusion that oral VGCV is effective as a preemptive treatment in all liver transplant recipients. However, the present study demonstrated that oral VGCV may prevent CMV disease in an outpatient setting and thereby reduce patient burden and health-care costs. When the first laboratory results suggesting CMV infection are detected, many liver transplant recipients without any signs and symptoms of CMV disease may benefit from outpatient treatment with oral VGCV.

## Supporting Information

S1 File Dataset and Sources. An Excel sheet containing information about the data used in this study.
(XLSX)
